# Anticanteen Meeting

**Published:** 1912-08

**Authors:** 


### Anticanteen Meeting.

On Monday afternoon, March 25th, a meeting was held, under the auspices of the W. C. T. U., in one of the ballrooms of the Hotel Astor, to discuss the objections to the restoration of the sale of beer and wine in the army post exchange or canteen. The speakers were military men who have seen both sides of the question—General Miles, and Colonel Maus, Chief Surgeon and Medical Inspector of the Central Division, U. S. Army. General Miles spoke of the desertions of the men in the anticanteen period, and showed from the army reports that desertions have been gradually lessening, 1911 showing the smallest percentage of desertions since the Civil War. He said beer is as injurious to soldiers as regards efficiency as it is to men in other lines of life.

Colonel Maus said that the increase in venereal diseases reported can be accounted for in two ways: First, there was not compulsory inspection until within the last decade; and, second, the personnel of the army has changed. The army now is made up of younger men who are naturally more passionate than the mature men who formerly served as soldiers. The strongest point made by Colonel Maus was that the young soldiers now in the army do not remain in the service more than a few years, and they know that if they learn to drink in the army it will hinder them from obtaining good positions in the business world when they return to civil life. So, many of them, he said, do not want the temptations of the beer canteen. He said he had permission from the Secretary of War to be present, that he had come 1000 miles to make this address, and he would gladly go ten times the distance to save the soldier boys from the army saloon.—*New York Medical Journal.*

